# Supplementary material for: Effects of Trichoderma harzianum combined with Phanerochaete chrysosporium on lignin degradation and humification during chicken manure and rice husk composting
Source: Front Microbiol. 2025 Feb 28;16:1515931. doi: 10.3389/fmicb.2025.1515931 (PMC11906335; doi:10.3389/fmicb.2025.1515931)
Supplement: Supplementary file 1 [file Data_Sheet_1.zip › 6.Function/CK0_BFS/FunGuild/l2/metastats/Animal_Pathogen.CK0_BFS.metastats.pdf]

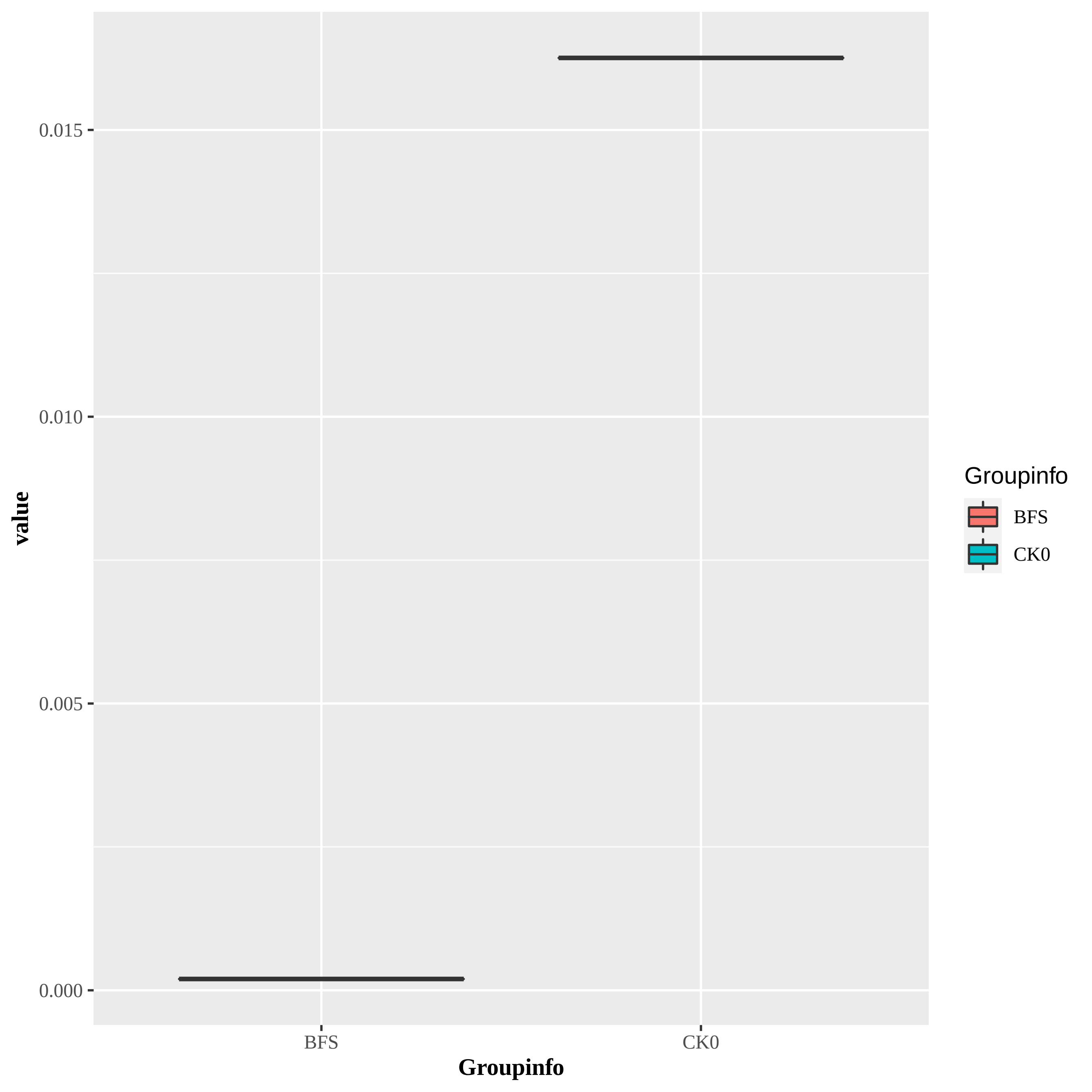

0.015
0.010
0.005
0.000
value
BFS
CK0
Groupinfo
Groupinfo
BFS
CK0
